# TO WHAT EXTENT IS THERE INTERGENERATIONAL CONTINUITY IN EARLY-LIFE STRESSORS?

**DOI:** 10.1093/geroni/igac059.579

**Published:** 2022-12-20

**Authors:** Olivia Atherton, Eileen Graham, Avron Spiro, Marc Schulz, Robert Waldinger, Daniel Mroczek, Lewina Lee

**Affiliations:** University of Houston, Houston, Texas, United States; Northwestern University, Chicago, Illinois, United States; Boston University, Jamaica Plain, Massachusetts, United States; Bryn Mawr College, Bryn Mawr, Pennsylvania, United States; Harvard University, Boston, Massachusetts, United States; Northwestern University, Chicago, Illinois, United States; Boston University School of Medicine, Boston, Massachusetts, United States

## Abstract

Prior work has investigated the correlates and consequences of early life stress within a person’s lifetime, but less is known about whether early life stressors are sustained across generations. Using multi-generational data from 1,312 offspring and their fathers (N = 518 families), we examined the extent to which there is intergenerational continuity in childhood social class, childhood home atmosphere, parent-child relationship quality, and childhood health, as well as whether person-level and family-level factors strengthen (or weaken) intergenerational continuity. Results suggest notable intergenerational continuity in childhood social class, but no continuity in childhood home atmosphere, parent-child relationship quality, or childhood health. Moreover, the intergenerational continuity of early life stressors was modified by father education level and education mobility, such that low education level conferred risks, and upward education mobility conferred benefits, for offspring adverse experiences. We discuss broader implications of the findings for future research, clinical interventions, and social policy.

